# Plant Electrophysiological Parameters Represent Leaf Intracellular Water–Nutrient Metabolism and Immunoregulations in *Brassica rapa* During *Plasmodiophora* Infection

**DOI:** 10.3390/plants14152337

**Published:** 2025-07-29

**Authors:** Antong Xia, Yanyou Wu, Kun Zhai, Dongshan Xiang, Lin Li, Zhanghui Qin, Gratien Twagirayezu

**Affiliations:** 1 Hubei Key Laboratory of Selenium Resource Research and Biological Application, Hubei Minzu University, Enshi 445000, China; tone1214910327@163.com (A.X.);; 2 State Key Laboratory of Environmental Geochemistry, Institute of Geochemistry, Chinese Academy of Sciences, Guiyang 550081, China; 3 Academy of Agricultural Sciences, Enshi Tujia and Miao Autonomous Prefecture, Enshi 445000, China

**Keywords:** electrophysiological parameters, *Plasmodiophora*, intracellular water–nutrient metabolism, immunoregulation, dielectric substance transfer

## Abstract

Although *Brassica rapa* (*B. rapa*) is vital in agricultural production and vulnerable to the pathogen *Plasmodiophora*, the intracellular water–nutrient metabolism and immunoregulation of *Plasmodiophora* infection in *B. rapa* leaves remain unclear. This study aimed to analyze the responsive mechanisms of *Plasmodiophora*-infected *B. rapa* using rapid detection technology. Six soil groups planted with Yangtze No. 5 *B. rapa* were inoculated with varying *Plasmodiophora* concentrations (from 0 to 10 × 10^9^ spores/mL). The results showed that at the highest infection concentration (PWB5, 10 × 10^9^ spores/mL) of *B. rapa* leaves, the plant electrophysiological parameters showed the intracellular water-holding capacity (IWHC), the intracellular water use efficiency (IWUE), and the intracellular water translocation rate (IWTR) declined by 41.99–68.86%. The unit for translocation of nutrients (UNF) increased by 52.83%, whereas the nutrient translocation rate (NTR), the nutrient translocation capacity (NTC), the nutrient active translocation (NAT) value, and the nutrient active translocation capacity (NAC) decreased by 52.40–77.68%. The cellular energy metabolism decreased with worsening *Plasmodiophora* infection, in which the units for cellular energy metabolism (∆G_E_) and cellular energy metabolism (∆G) of the leaves decreased by 44.21% and 78.14% in PWB5, respectively. Typically, based on distribution of B-type dielectric substance transfer percentage (BPn), we found PWB4 (8 × 10^9^ spores/mL) was the maximal immune response concentration, as evidenced by a maximal BPn_R_ (B-type dielectric substance transfer percentage based on resistance), with increasing lignin and cork deposition to enhance immunity, and a minimum BPn_Xc_ (B-type dielectric substance transfer percentage based on capacitive reactance), with a decreasing quantity of surface proteins in the *B. rapa* leaves. This study suggests plant electrophysiological parameters could characterize intracellular water–nutrient metabolism and immunoregulation of *B. rapa* leaves under various *Plasmodiophora* infection concentrations, offering a dynamic detection method for agricultural disease management.

## 1. Introduction

*Brassica rapa* (*B. rapa*) is a commonly grown *Brassica* crop in Asia. The cultivated area in China represents at least 15% of the total vegetable cultivation area of the country, and the cultivation method and nutritional value of *B. rapa* have long been widely recognized [1,2]. Unfortunately, it can be infected by *Plasmodiophora*, one of the most severe soil-borne pathogens of *Brassica* plants, caused by the flagellate fungus *Plasmodiophora brassicae*, which mainly attacks plant roots [3]. After a severe attack caused by *Plasmodiophora*, plants exhibit several symptoms, including the expansion and swelling of root structures, impeded development of above-ground parts [4], slowed growth [3], and even death [4]. Therefore, it is crucial to reveal the effects of *Plasmodiophora* infection on *B. rapa.*

The previously established methods for assessing plant growth, such as Koch’s law [5], enzyme immunoassays [6], and multispectral imaging [7], have drawbacks, including time consumption, operational difficulty, etc. Additionally, these methods damage plant tissues, making it challenging to quantify the dynamic changes in plant growth. Therefore, a suitable methodology that can analyze the dynamic changes in *B. rapa* growth during *Plasmodiophora* infection without causing damage is urgently needed.

Compared with other methods, measuring plant electrical signals is the best technique for performing plant electrophysiological analysis, providing an excellent alternative. The main physiological parameters of plants include capacitance (C), resistance (R), impedance (Z), capacitive reactance (X_C_), and inductive reactance (X_L_). When plants are affected by metabolic alterations and external stresses, they produce electrical responses, which are changes in the potentials generated by cells and tissues [8]. The cell membrane, which consists of a phospholipid bilayer (PLB) and internal compounds (lipids, proteins, sugars, etc.), is the crucial site for generating plant electrical signals [9]. Typically, the phospholipid bilayer can be divided into electron-dense bands on both the inner and outer membrane sides and a transparent band in the middle. The bilayer structure of the cell membrane is the source of the electrical properties of a cell, and membrane lipids can be viewed as an insulating layer having high electrical resistivity, which induces the cell to store charges [10]. The cell membrane has strict selective permeability to ions, and the electrolyte solution (ES) on both sides has a specific conductive state [11]. The inner and outer sides of the membrane can be modeled as a leaky capacitor (LC). The solution on both sides of the membrane can be viewed as the two polar plates of the capacitor [12]. The cell membrane acts as the dielectric medium of the capacitor [13]. The intracellular ions, ionic groups (IGs), and electric dipoles (EDs) are equivalent to the electrolytes in which plant cell membranes are capacitive [14]. When adverse conditions damage cells, their structure, composition, and ion permeability alter, leading to changes in the electrical properties [15].

Recently, electrophysiology technology has been widely used to analyze the growth of potatoes [14], tomatoes [16], wheat [17], and other crops, as well as in disease prevention. In addition, it is used in the analysis of dynamic changes in plant growth, a process accompanied by the water, nutrient, and cellular metabolism of plant species [16]. All these processes involve charge separation, electron movement, and the transport of dielectric substances, leading to changes in plant electrical signals [17]. Similarly, when plants are subjected to external stimuli, electrical signals provide direct feedback on changes in growth, which are reflected in changes in photosynthesis, water and nutrient uptake, and cell metabolic energy [18]. Since the substance transport capacity depends on the type and amount of surface and bound proteins in the cell membrane [19], the cell membrane structure can maintain the stability of the cellular environment by facilitating the transfer of substances through intrinsic and extrinsic proteins. The plant’s cell membrane chiefly determines its electrical resistance, with capacitance being influenced by the variety and number of extrinsic proteins and inductance being affected by the variety and number of intrinsic proteins [20]. Typically, ∆G and BPn promote the dynamic growth of plant species. Therefore, based on the determined R, Xc, and X_L_, three types of B-type dielectric substance transfer percentage—which are BPn_R_ (the B-type dielectric substance transfer percentage based on resistance), BPn_Xc_ (the B-type dielectric substance transfer percentage based on capacitive reactance), and BPn_XL_ (the B-type dielectric substance transfer percentage based on inductive reactance)—need to be determined.

This study aimed to assess the performance of electrophysiological techniques for detecting *Plasmodiophora* infection in *B. rapa* by analyzing its growth status, photosynthesis, and electrical signals. It also investigated (1) the electrophysiological responses of *B. rapa* to *Plasmodiophora* and (2) the synergistic responses of intracellular water metabolism, nutrient translocation, ATP metabolism, and substance transfer characterization at different levels of *Plasmodiophora* infection in *B. rapa*, with the primary purpose of preventing *Plasmodiophora* infection in agricultural and food production.

## 2. Materials and Methods

### 2.1. Plant Selection and Preparation of Conidial Suspensions

In this study, Yangtze No. 5 *B. rapa*, which is abbreviated as *B. rapa* in this experiment, sourced from Dezhou City, Shandong Province of China, was chosen for the experiments because of its notable strong disease resistance. *Plasmodiophora* was selected because it is a prevalent fungal pathogen that commonly infects *B. rapa* in China. *Plasmodiophora* was extracted meticulously following the detailed steps a-f outlined in Figure S1 (the process of infection with *Plasmodiophora*).

### 2.2. Plasmodiophora Infection Treatment

**Figure 1:**
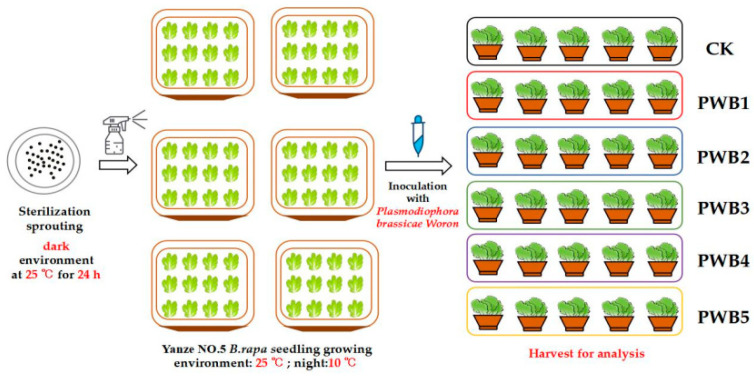
The infection experiment of Plasmodiophora on *B. rapa* at different concentrations as follows: 0, 2 × 10^9^, 4 × 10^9^, 6 × 10^9^, 8 × 10^9^, and 10 × 10^9^ spores/mL.

Figure 1 illustrates the infection of *B. rapa* with various concentrations of *Plasmodiophora*. The infected seeds were sown in hole trays containing sterile soil and cultivated under controlled conditions, with temperatures maintained at 25 °C during the day and 10 °C at night. On the 30th day, the seedlings were subjected to treatments with *Plasmodiophora* at six different concentrations: 0, 2 × 10^9^, 4 × 10^9^, 6 × 10^9^, 8 × 10^9^, and 10 × 10^9^ spores/mL. Following this treatment, samples were harvested after 14 days to assess the impact of fungal infection.

### 2.3. Biomass Estimation

The root-to-shoot ratio of *B. rapa* (Equation (1)) under the various treatments was quantified by initially heating the root, stem, and leaf parts of the plants at 108 °C for 30 min, and then drying them at 70 °C.

R/S (%) = Biomass (Root)/Biomass (Shoot) × 100%(1)

where R represents the root, and S denotes the above-ground parts (shoot) of the plant.

### 2.4. Measurement of Photosynthesis of Plant Species

The 2nd and 3rd fully expanded leaves of *B. rapa* were used to measure photosynthesis from 9:00 to 11:00 a.m. using the LI-6400 photosynthesis system (LI-COR, Lincoln, NE, USA). Herein, the net photosynthetic rate (Pn, μmol/m^2^·s^−1^), stomatal conductance (Cond, mmol/H_2_O m^2^·s^−1^), transpiration rate (Tr, mmol H_2_O/m^2^·s^−1^), and intercellular CO_2_ concentration (Ci, μmol CO_2_ mol^−1^·air) were determined. Water use efficiency (WUE) was calculated using Equation (2). The other measured parameters included a temperature of 25 °C, a CO_2_ concentration of 400 μmol mol^−1^ in buffered glass bottles, and a photosynthetically active radiation intensity of 500 μmol/m^2^·s^−1^.

WUE (%) = Pn/Tr × 100%(2)

### 2.5. Measurement of Electrophysiological Parameters of the Plant

**Figure 2:**
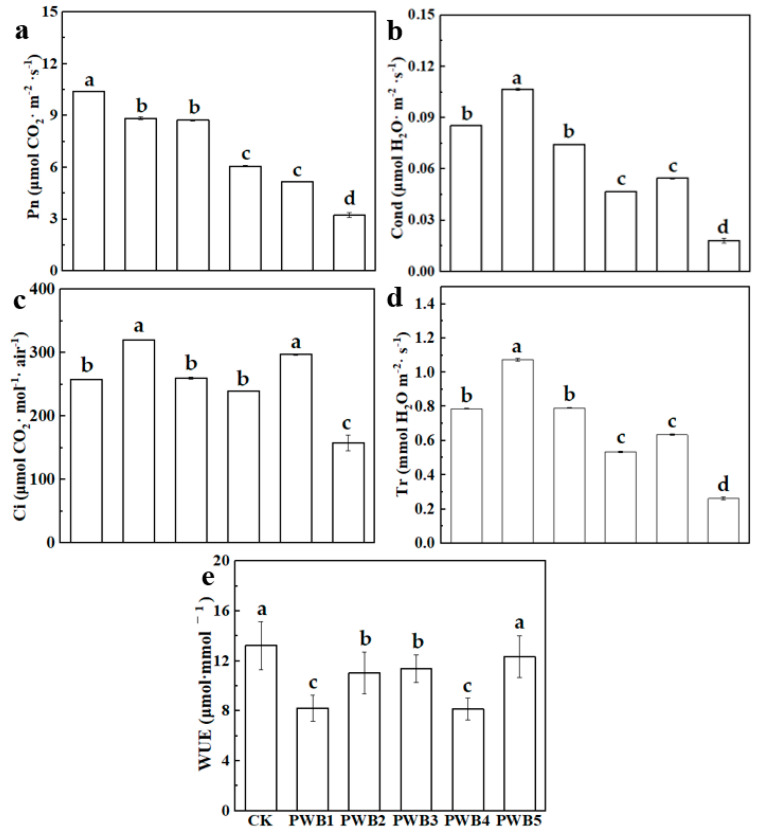
Photosynthesis of *B. rapa* at different concentrations of *Plasmodiophora* infection. The data in the graph are presented as the mean ± standard deviation, n = 5; n is the number of plants per treatment. PWB denotes the infection by *Plasmodiophora* of *B. rapa* at different concentrations as follows: CK-0, PWB1-2 × 10^9^, PWB2-4 × 10^9^, PWB3-6 × 10^9^, PWB4-8 × 10^9^, and PWB5-10 × 10^9^ spores/mL. (**a**): Pn photosynthetic rate; (**b**): Cond indicates stomatal conductivity; (**c**): Tr indicates transpiration; (**d**): Ci indicates intercellular carbon dioxide concentration; and (**e**): WUE indicates water use efficiency. The different lowercase letters a, b, and c in the table denote the significance of differences at *p* < 0.05.

Since photosynthesis in leaves does not directly reflect electrophysiological parameters of the experiment, the second fully expanded leaf of each plant was selected to measure the electrophysiological parameters (Figure 2). The experimental environment was as follows: air relative humidity (75 ± 5)% and daytime/night cycle temperature (30 °C/20 °C). The leaves were placed between two electrode plates (silver electrode, Ag) of a parallel-plate capacitor, which was operated by an LCR-6100 LCR meter (Gwinstek, Taiwan, China) with the frequency (f) set to 3 kHz and the voltage (U) set to 1.5 V. The measurement equipment included a holder, electrode plates, wires, iron blocks, and plastic rods. The electrode plates were embedded in the bracket and the bottom of the plastic rods (Figure S2) and were connected to the LCR meter through wires. The two electrode plates clamped the leaves to be measured. In the parallel mode, C, R, and Z of the plant leaves were determined by adding different numbers of iron blocks of equal mass with a fixed clamping force (1 N, 2 N, 3 N, 5 N, and 7 N). R and Z were measured 15 times for each plant leaf, and we established fitting equations for the electrophysiological parameters.

### 2.6. Calculation of Plant Electrophysiological Parameters

Considering the metabolic energy of plant cells as a factor, the application of different clamping forces alters the structural morphology of the chloroplasts, thereby affecting the electrical signaling activities within the cells. Based on the Gibbs free energy equation and the Nernst equation, this study constructed a model to describe the variations in R, Z, and C of the plant leaves with clamping force, as shown in Equation (3). The steps for deriving C, R, Z, Xc, and X_L_ of the plant leaves are detailed in Equations (4)–(8). The variation in plant leaf X_L_ with clamping force was modeled as Equation (9).

(3) $$F=\left(M+m\right)g$$

(4) $$C=x_{0}+hF$$

(5) $$R=y_{0}+k_{1}e^{-b_{1}F}$$

(6) $$Z=p_{0}+k_{2}e^{-b_{2}F}$$

(7) $$Xc=q_{0}+k_{3}e^{-b_{3}F}$$

(8) $$\frac{1}{-X_{L}}=\frac{1}{Z}-\frac{1}{R}-\frac{1}{Xc}$$

(9) $$X_{L}=a_{0}+k_{4}e^{-b_{4}F}$$

where F is the clamping force exerted by the iron block in newtons (N), M is the mass of the iron block (kg), m is the mass of the plastic rod and the electrode sheet (kg), g is acceleration due to gravity (9.8 N/kg), C is the capacitance of the plant leaves, $x_{0}$ is a constant, h is a constant, R is the resistance of the plant leaves, $y_{0}$ is a constant, $k_{1}$ is a constant, $b_{1}$ is a constant, e is the base of the natural logarithm, Z is the impedance of the plant leaves, p_0_ is a constant, $k_{2}$ is a constant, $b_{2}$ is a constant, F is the clamping force, e is the base of the natural logarithm, Xc is a capacitive reactance of the plant leaves, q_0_ is the initial capacitive reactance without any clamping force, k_3_ is a constant relating exponential decay to the clamping force, b_3_ is a decay constant for the capacitive reactance concerning clamping force, X_L_ is the inductive reactance of the plant leaves, R is the resistance of the plant leaves, Xc is the capacitive reactance of the plant leaves, a_0_ is the initial inductive reactance without any clamping force, k_4_ is a constant relating exponential decay to the clamping force, and b_4_ is a decay constant for the inductive reactance concerning clamping force.

As the nutrient capacity of plant leaves can be calculated when F is 0, we obtain intrinsic resistance (IR), the intrinsic capacitive reactance (IXc), and the intrinsic inductive reactance (IX_L_) using Equations (10)–(12), and intrinsic impedance (IZ) and intrinsic capacitance (IC_P_) were calculated using Equations (13) and (14).

(10) $$IR=y_{0}+k_{1}$$

(11) $$IXc=q_{0}+k_{3}$$

(12) $$IX_{L}=a_{0}+k_{4}$$

(13) $$\frac{1}{IZ}=\frac{1}{IR}+\frac{1}{IXc}-\frac{1}{IX_{L}}$$

(14) $$IC_{P}=\frac{1}{2\pi fIX_{L}}$$

where y_0_ is the initial or baseline resistance value, k_1_ is a constant or coefficient that modifies the initial resistance, q0 is the initial or baseline capacitive reactance value, k3 is a constant or coefficient that modifies the initial capacitive reactance, a0 is the initial or baseline inductive reactance value, k4 is a constant or coefficient that modifies the initial inductive reactance, and f is frequency.

### 2.7. Estimation of Intracellular Water Metabolism in Plants Based on Electrophysiological Information

Based on the water-holding capacity of cells, which is directly proportional to C3/2, it is possible to characterize the water-holding capacity of the plant leaves using Equation (15) (IWHC). The specific adequate thickness (d) of the plant leaves was calculated using Equations (16) and (17). The relative intracellular water use efficiency (IWUE), intracellular water-holding time (IWHT), and water transfer rate (WRT) of the plant leaves were obtained using Equations (18)–(20).

(15) $$IWHC=\sqrt{{IC_{p}}^{3}}$$

(16) $$k_{0}=\frac{U^{2}}{2d}$$

(17) $$d=\frac{U^{2}h}{2}$$

(18) $$IWUE=\frac{d}{IWHC}$$

(19) IWHT=C×Z

(20) $$WRT=\frac{IWHC}{IWHT}$$

### 2.8. Nutrient Transport Capacity Based on Electrophysiological Information Characterization

Based on the plant electrophysiological information, the unit for nutrient-relative transport (UNF), the nutrient transfer rate (NTR), nutrient transfer capacity (NTC), the unit for nutrient active flow (UAF), and nutrient active transport capacity (NAC) were calculated using Equations (21)–(25).

(21) $$UNF=\frac{R}{IXc}+\frac{R}{IX_{L}}$$

(22) $$NTR=\frac{IWHC}{IWHT}$$

(23) NTC=UNF×NTR

(24) $$UAF=\frac{IX_{C}}{{IX}_{L}}$$

(25) NAC=UAF×NTR

where R represents resistance, IXc is the current flow through a cell, IX_L_ is the current flow through a leaf, IWHC is the integrated water-holding capacity, IWHT is the integrated water-holding time, UNF is a nutrient-relative transport unit, NTR is the nutrient transfer rate, UAF is a nutrient active flow unit, and NTR is the nutrient transfer rate.

### 2.9. The Cellular Metabolic Energy for B. rapa Leaves

Based on C, R, and Z of the plant leaves, cellular metabolic energy can be calculated based on a model constructed with electrophysiological parameters. According to the parameters from Equation (4), the unit for the metabolizable energy of leaf cells (ΔG_R−E_) can be obtained as shown in Equation (26). Based on Equation (5), the Z-based metabolic energy per unit of plant leaf cells (ΔG_Z−E_) can be obtained using Equation (27). The cellular energy metabolism for R (ΔG_R−E_) and Z (ΔG_Z−E_) and the average metabolic energy (ΔG) were calculated using Equations (28)–(30).

(26) $$\Delta G_{R-E}=\frac{lnk_{1}-lny_{0}}{b_{1}}$$

(27) $$\Delta G_{Z-E}=\frac{lnk_{2}-lnp_{0}}{b_{2}}$$

(28) $$\Delta G_{R}=\Delta G_{R-E}d$$

(29) $$\Delta G_{Z}=\Delta G_{Z-E}d$$

(30) $$\Delta G=\frac{\Delta G_{R}+\Delta G_{Z}}{2}$$

where lnk_2_ is a natural logarithm of the rate constant k_2_, lnp_0_ is the natural logarithm of the initial parameter p_0_, b_2_ is a coefficient related to energy calculation, ΔG_R_ is a metabolic energy related to parameter R, ΔG_R−E_ is energy per unit related to parameter R, d is a dimensionless scaling factor, ΔG_Z_ is metabolic energy related to parameter Z, ΔG_Z−E_ is energy per unit related to parameter Z, ΔG is average metabolic energy, ΔG_R_ is metabolic energy associated with R, and ΔG_Z_ is metabolic energy related to Z.

### 2.10. B-Type Dielectric Substance Transfer Percentage of B. rapa Leaves

The B-type dielectric substance transfer percentage is represented by Equations (31)–(34). The percentage values for the R, Xc, and X_L_ components of the B-type dielectric substance transfer percentage are detailed in Equations (35)–(37). Here, BPn_R_, BPn_Xc_, and BPn_XL_ represent the B-type dielectric substance transfer percentage associated with R, Xc, and X_L_; Bn_T_ represents the total transfer number, which is the sum of BPn_R_, BPn_Xc_, and BPn_XL_.

(31) $${Bn}_{R}=b_{1}$$

(32) $${Bn}_{Xc}=b_{3}$$

(33) $${Bn}_{Xl}=b_{4}$$

(34) $${Bn}_{T}={Bn}_{R}+{Bn}_{Xc}+{Bn}_{Xl}$$

(35) $${BPn}_{R}=\frac{100{Bn}_{R}}{{Bn}_{T}}\%$$

(36) $${BPn}_{Xc}=\frac{100{Bn}_{Xc}}{{Bn}_{T}}\%$$

(37) $${BPn}_{Xl}=\frac{100{Bn}_{Xl}}{{Bn}_{T}}\%$$

### 2.11. Statistical Analysis

The statistical software SPSS 25.0 was employed to analyze variance (ANOVA) and perform Duncan’s multiple range tests to identify significant differences. Additionally, SPSS was utilized to conduct Pearson correlation analysis to determine the relationship between each indicator [10,15]. The experimental results are presented as the mean ± standard deviation (Mean ± SE). The graphs and figures were generated using Origin 2021Pro.

## 3. Results

### 3.1. Plant Biomass

Table 1 illustrates the biomass of *B. rapa* after infection with varying concentrations of *Plasmodiophora*. As the concentration of *Plasmodiophora* increased, there was a corresponding decrease in the root, shoot, and total biomasses of the plants. Notably, the reduction in biomass was most pronounced in *B. rapa* treated with PWB5. Specifically, the root biomass decreased by 74.93%, the shoot biomass by 26.38%, and the total biomass by 71.24% compared with the control group (CK). Despite the overall biomass reduction, the root–shoot ratio (R/S) of PWB5-treated *B. rapa* significantly increased by 50.45% compared with that of the CK.

### 3.2. Photosynthesis in Plants

Figure 2 illustrates the impact of varying concentrations of *Plasmodiophora* on the photosynthesis-related parameters in *B. rapa*. As shown in Figure 2a, the photosynthetic rate (Pn) significantly decreased with increasing *Plasmodiophora* concentrations. PWB5 exhibited the lowest Pn, approximately 68.87% less than that of CK. This declination indicates a dose-dependent adverse effect of *Plasmodiophora* on the photosynthetic efficiency of *B. rapa*. As depicted in Figure 2b, similar to Pn, the level of stomatal conductivity (Cond) decreased as the R. solami concentration increased. The CK group maintained the highest Cond level, whereas PWB5 had the lowest, with a reduction of 78.66%. This indicates impaired stomatal function and gas exchange due to *Plasmodiophora* infection. Figure 2c shows a substantial decrease in the transpiration rate (Tr) with higher *Plasmodiophora* levels. PWB5 exhibited the lowest Tr, with a reduction of 66.68% compared with the control. The decreased Tr reflects impaired water movement and possible stomatal closure due to infection. Figure 2d shows that the intercellular CO_2_ concentration (Ci) decreases as the *Plasmodiophora* concentration increases. CK has the highest Ci, while PWB5 has the lowest, with a 38.78% decrease compared to CK. This reduction in Ci suggests restricted CO_2_ diffusion into the leaf, impacting photosynthesis. As represented in Figure 2e, water use efficiency (WUE) exhibited the lowest value in the PWB4 treatment, not PWB5. PWB4 showed a 38.32% decrease in WUE compared with that of CK. However, the water usage efficiency (WUE) in PWB5 did not significantly differ from that of CK.

### 3.3. Fitting Equations for Leaf Electrophysiological Parameters and Clamping Force

**Figure 3:**
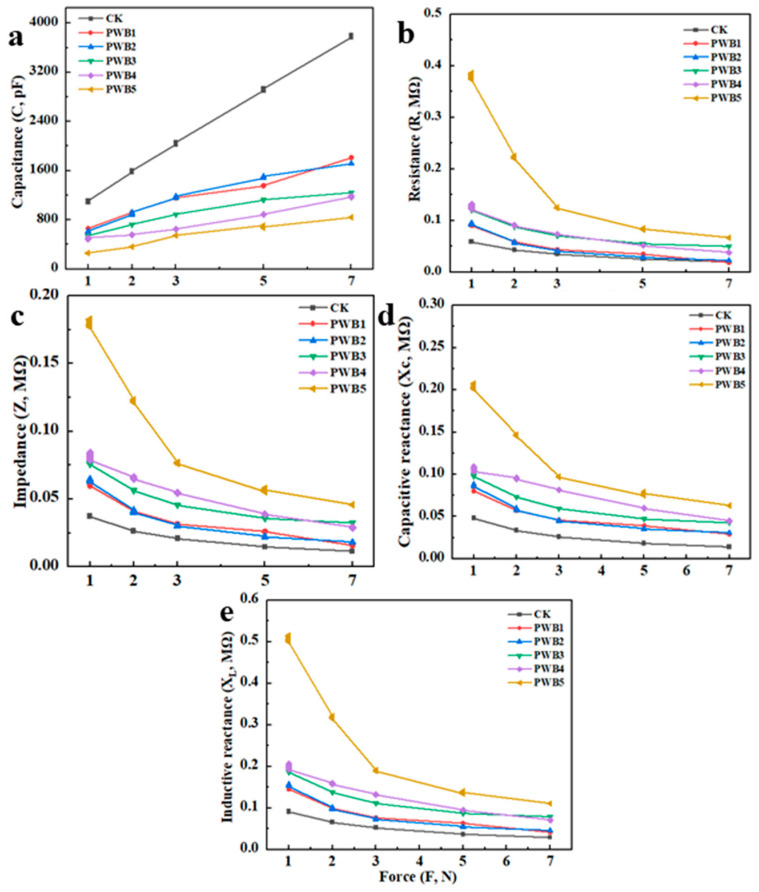
Fitting equations for electrophysiological parameters of *B. rapa* leaves in response to different clamping forces. *Plasmodiophora* concentrations were CK-0, PWB1-2 × 10^9^, PWB2-4 × 10^9^, PWB3-6 × 10^9^, PWB4-8 × 10^9^, and PWB5-10 × 10^9^ spores/mL. C indicates capacitance (**a**), R indicates resistance (**b**), Z indicates impedance (**c**), Xc indicates capacitive reactance (**d**), and X_L_ indicates inductive reactance (**e**). R^2^ indicates that correlation of fitted equations is 0.99, and *p* < 0.01 indicates significance difference at 0.01.

Table 2 and Figure 3 indicate the fitted equations of the electrophysiological parameters and the clamping force of *Plasmodiophora*-infected *B. rapa.* In this study, the C, R, Z, X_C_, X_L_, and F of *B. rapa* showed robust significance (R^2^ = 0.99, *p* < 0.01), proving that the phytoelectric signals could reliably characterize the response of *B. rapa* to infection with varying levels of *Plasmodiophora*. The C of the *B. rapa* leaves showed a trend opposite to that of R, Z, X_C_, and X_L_, with C showing a significant positive correlation with F. In contrast, R, Z, X_C_, and X_L_ showed a marked negative association with F. C, Z, X, and F were remarkably correlated with F. In contrast, R, Z, X_C_, and X_L_ were negatively associated with F.

### 3.4. Electrophysiological Information About Plants During Plasmodiophora Infection

Based on the fitting equations of *B. rapa* electrical signals to the clamping force (F) after infection with varying concentrations of *Plasmodiophora*, the present study deduced the intrinsic electrophysiological information about the *B. rapa* leaves when F = 0 (Table 3). The IC_P_ of *B. rapa* decreased with increasing *Plasmodiophora* contents, whereas IR, IZ, IX_C_, and IX_L_ increased. The IC_P_ of the PWB5 variety was the smallest, being 78.09% lower than that of the CK, whereas the IR, IZ, IX_C_, and IX_L_ values were approximately 4–8 times higher than those of the CK. Specifically, the IR of PWB5 was 67.45 ± 10.00 MΩ, which was significantly higher than the CK value of 8.75 ± 1.25 MΩ. The IZ value for PWB5 was 29.69 ± 0.82 MΩ, whereas the CK had a value of 5.59 ± 0.44 MΩ. The IXC for PWB5 reached 33.25 ± 1.57 MΩ compared to 7.28 ± 0.29 MΩ for the CK. Similarly, the IX_L_ value of PWB5 was 87.71 ± 7.99 MΩ, markedly higher than the CK’s 13.70 ± 1.35 MΩ. The trend observed indicates a significant electrophysiological shift in *B. rapa* with increased *Plasmodiophora* concentration.

### 3.5. Intracellular Water Metabolism Based on Plant Electrical Signal Quantification in B. rapa

The intracellular water metabolism characteristics of *B. rapa* were determined using the intrinsic electrophysiological data collected after the plant was infected with various concentrations of *Plasmodiophora* (Table 4). This study revealed that the IWHC, IWUE, and WRT of *B. rapa* decreased, whereas the IWHT increased with increased *Plasmodiophora* concentrations. The IWHC and the WRT were the lowest in PWB5, where they were 63.66% and 68.86% less than those of the CK, respectively. Specifically, the IWHC decreased from 8102.65 ± 211.46 in the CK to 2944.35 ± 92.05 in PWB5, and the WRT decreased from 199.54 ± 12.54 in the CK to 62.13 ± 2.30 in PWB5. Conversely, the IWHT was the highest in PWB4, showing a 17.83% increase compared with the CK, with the values rising from 40.67 ± 1.60 in the CK to 47.92 ± 1.26 in PWB5. Additionally, IWUE was the lowest in PWB3, showing a 55.64% reduction compared to the CK, with the values decreasing from 6.74 ± 1.11 in the CK to 2.99 ± 0.80 in PWB3. The other treatments also showed significant changes, with varying degrees of decrease in the IWHC, IWUE, and the WRT, whereas the IWHT increased with the concentration of *Plasmodiophora*.

### 3.6. Characterization of Nutrient Transport in B. rapa Plant

The nutrient transport characteristics of *B. rapa* were obtained based on the intrinsic electrophysiological information about *B. rapa* after infection with different *Plasmodiophora* concentrations (Table 5). It was identified that the UNF of *B. rapa* was significantly enhanced post-infection with worsening *Plasmodiophora* infection, with the UNF value of PWB5 being the highest at 280.48 ± 42.46 × 10^−2^, indicating a 52.83% increase compared with the CK. Conversely, the NTR, NTC, UAF, and NAC values were reduced as the *Plasmodiophora* concentration increased. Specifically, the NTR of PWB5 decreased to 62.13 ± 2.30, which is a reduction of 68.86% compared to the CK. The NTC of PWB5 fell to 173.70 ± 20.94, representing a 52.40% decrease from the CK value. Similarly, the UAF of PWB5 showed a 28.36% reduction, and the NAC value of PWB5 significantly diminished to 23.82 ± 4.21, showing an 77.68% decrease compared to the CK. These findings highlight the adverse impact of higher *Plasmodiophora* concentrations on the nutrient transport efficiency in *B. rapa.*

### 3.7. Cellular Metabolic Energy for the Leaf of B. rapa in Response to Plasmodiophora

**Figure 4:**
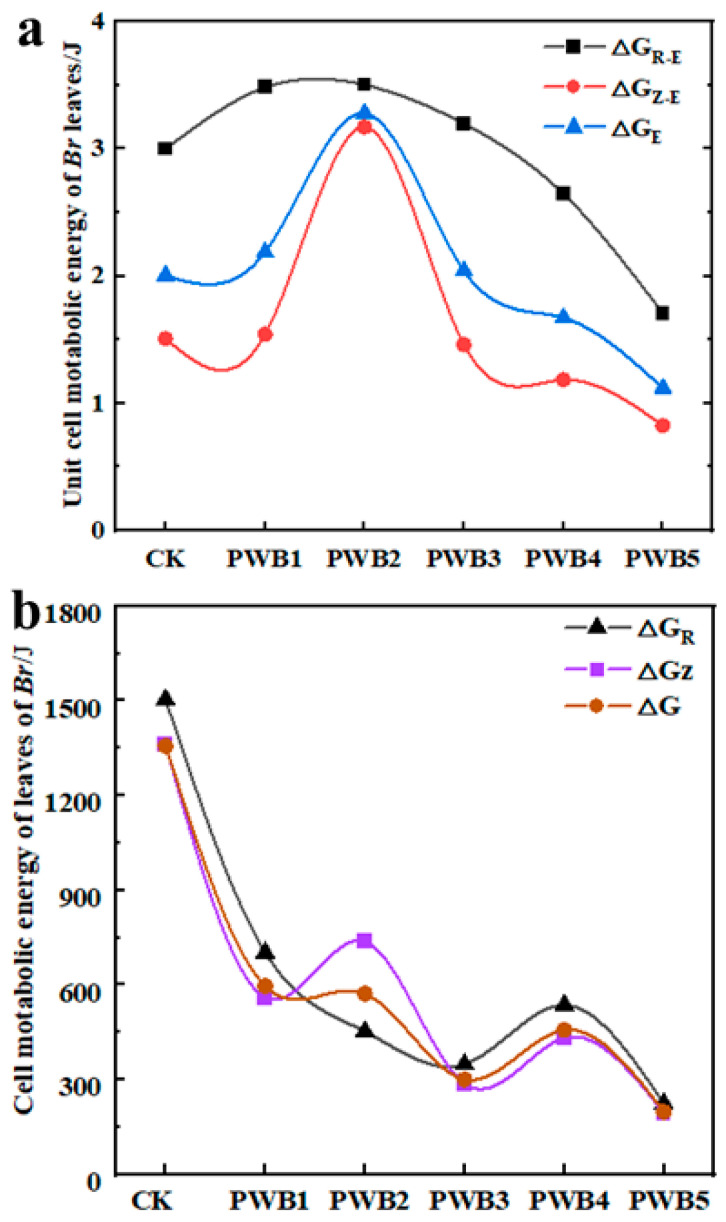
Unit for cell energy metabolism (**a**) and cell energy metabolism (**b**) of leaves in *B. rapa.* PWB represents effect of *Plasmodiophora* infection on *B. rapa*; different *Plasmodiophora* treatment concentrations were CK-0, PWB1-2 × 10^9^, PWB2-4 × 10^9^, PWB3-6 × 10^9^, PWB4-8 × 10^9^, and PWB5-10 × 10^9^ spores/mL. In (**a**), ∆G_R-E_, ∆G_Z-E_, and ∆G_E_ represent units for cell metabolizable energy for R, Z, and chloroplast of *B. rapa*, respectively. In (**b**), ∆G_R_, ∆G_Z_, and ∆G_E_ represent total cell metabolizable energy for R, Z, and chloroplast of *B. rapa*, respectively.

Figure 4 illustrates the unit for metabolic energy (a) and the total metabolic energy (b) for the leaves of *B. rapa* in response to *Plasmodiophora* infection. As indicated in Figure 4a, the unit for cell metabolizable energies for R and Z denoted as ∆G_R-E_, ∆G_Z-E_, and ∆G_E_ displayed varying trends of increase and decrease. Compared with those in the CK, ∆G_R-E_ and ∆G_E_ at PWB1 peaked, showing rises of 14.20% and 13.29%, respectively. Conversely, at PWB3, these values dropped to their lowest, with decreases of 33.11% and 35.22%, respectively. As shown in Figure 4b, ∆G_R_, ∆G_Z_, and ∆G_E_ of the *B. rapa* leaves generally declined with worsening *Plasmodiophora* infection. Although there were minor increases at PWB2 and PWB4, they remained lower than the CK. The minimum values were observed at PWB5, with reductions of 77.10% and 79.26% for ∆G_R_, ∆G_Z_, respectively, compared with the CK.

### 3.8. B-Type Dielectric Substance Transfer Percentage in B. rapa Leaves

**Figure 5:**
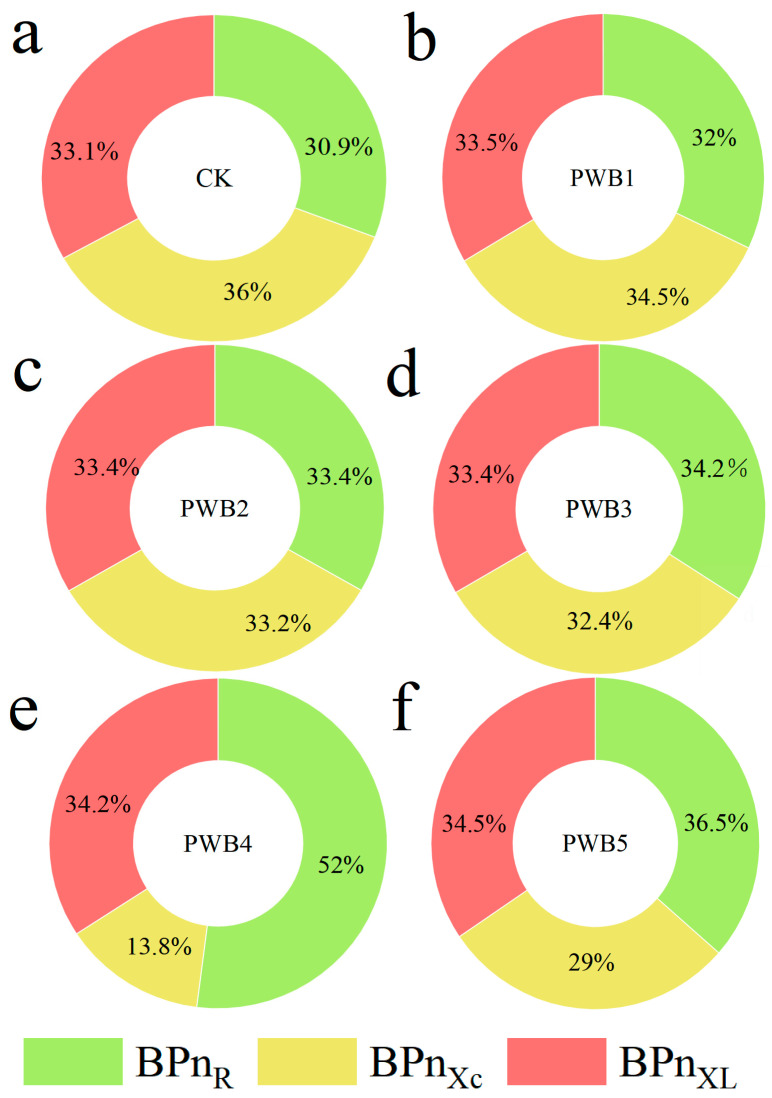
B-type dielectric substances transfer percentage of *B. rapa* at different *Plasmodiophora* infection concentrations. Different *Plasmodiophora* treatment concentrations are represented by CK-0 (**a**), PWB1-2 × 10^9^ (**b**), PWB2-4 × 10^9^ (**c**), PWB3-6 × 10^9^ (**d**), and PWB4-8 × 10^9^ (**e**), and PWB5-10 ×10^9^ (**f**) spores/mL. BPn_R_, BPn_Xc_, and BPn_XL_ represent percentage of B-type dielectric coefficients based on R, Xc, and X_L_ in *B. rapa.*

Figure 5 illustrates the percentage of B-type dielectric coefficients based on R, Xc, and X_L_ in the *B. rapa* leaves, denoted as BPn_R_, BPn_Xc_, and BPn_XL_. The analysis shows that as the degree of *Plasmodiophora* infection increases, these dielectric coefficients show a noticeable change. Specifically, PWB4 exhibited the highest BPn_R_, approximately 21.1% higher than that of the CK. Conversely, the BPn_Xc_ values for all the treatments were lower than those of the control, with PWB4 showing the lowest BPn_Xc_, which was 22.2% lower than that of the CK.

### 3.9. Correlation Between Growth and Electrophysiological Information About B. rapa During Plasmodiophora Infection

**Figure 6:**
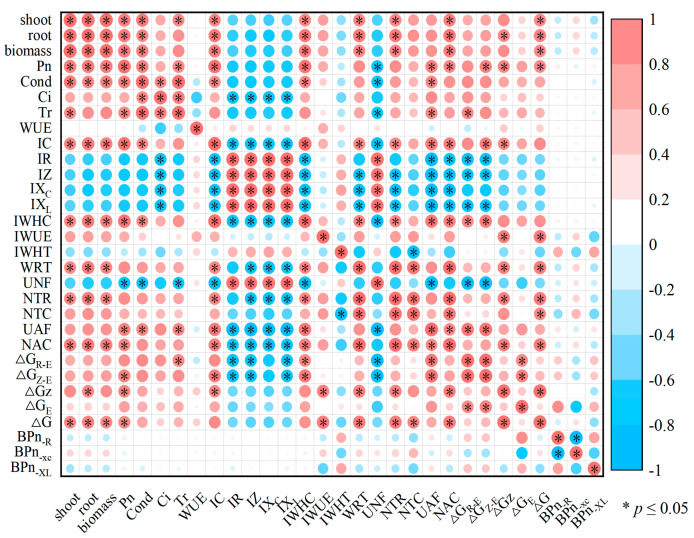
Correlation between growth, electrophysiological information, cellular metabolizable energy, and dielectric substance transfer capacity of *B. rapa* at different *Plasmodiophora* infection levels. Biomass denotes drying weight of *B. rapa*, Pn denotes net photosynthetic rate, IC denotes intracellular capacitance, IR denotes intracellular resistance, IZ denotes intracellular impedance, IXc denotes intracellular capacitive reactance, IX_L_ denotes intracellular reactance, IWHC denotes intracellular water-holding capacity, IWUE denotes intracellular water use efficiency, IWHT denotes intracellular water-holding time, WRT denotes water rate translocation, UNF denotes unit for translocation of nutrients, NTR denotes nutrient translocation rate, NTC denotes nutrient translocation capacity, UAF denotes nutrient active flow, NAC denotes nutrient active translocation capacity, ΔG_E_ denotes unit of cellular metabolic energy, ΔG denotes total of cellular metabolic energy, BPn_R_ denotes B-type dielectric material transfer capacity of R, BPn_Xc_ denotes B-type dielectric material transfer capacity of X_C_, and BPn_XL_ denotes B-type dielectric material transfer capacity of X_L_. ‘*’ represents significant correlation at 0.05 level.

Figure 6 illustrates the correlation between growth, electrophysiological parameters, cellular metabolizable energy, and dielectric substance transfer capacity of *B. rapa* at varying levels of *Plasmodiophora* infection. The analysis showed that the growth of *B. rapa* was significantly positively correlated with several factors, including biomass, Pn, IC_P_, IWHC, IWUE, the NTR, NAC, and ΔG. Conversely, growth was significantly negatively correlated with IR, IZ, IXc, IX_L_, and the UNF.

## 4. Discussion

### 4.1. Electrophysiology Can More Sensitively Reflect Plasmodiophora Infection

In this study, we found *Plasmodiophora* infection affected the dynamic plant electrical signals of *B. rapa*. Equations for calculating C, R, Z, X_C_, and X_L_ of the *B. rapa* leaves were established under a fixed clamping force (Equations (3)–(9)). The correlation coefficient (R^2^) between C, R, Z, X_C_, X_L_, and F of the *B. rapa* leaf blades was 0.99, and the *p*-values of all the parameters of the fitted equations were <0.0001 (Table 4). These results indicate a significant correlation between the clamping force and the electrical signals (C, R, Z, X_C_, and X_L_; Figure 5) [21]. Therefore, when F was 0, we calculated the intrinsic electrical signals (IC_P_, R, Z, X_C_, and X_L_) of the *B. rapa* leaves (Table 3); the changes in growth conditions (Table 1) and photosynthesis (Figure 2a–d) of *B. rapa* were directly proportional with capacitance, but inversely proportional with resistance, impedance, capacitance, and susceptibility. These findings indicate that the growth of *B. rapa* was inversely proportional to the degree of *Plasmodiophora* infection, which could be attributed to a reduction in the biomass and photosynthetic capacity of *B. rapa* in previous research. When elm trees were infected with *Fusarium* oxysporum, the electrical resistance of the trunks was substantially elevated, but their growth metabolism was undermined [22]. The electrical resistance of fir trees infected with sooty mold was found to have a significant negative correlation with their growth status [23,24]. The impedance of the root system of apple trees elevated conspicuously with susceptibility to disease [25,26]. The growth of disease-infected apple trees was inhibited, suppressing the electrical signals [27,28].

### 4.2. Plasmodiophora Infection Changed Intracellular Water and Nutrient Metabolism of B. rapa Leaves

The intracellular water and nutrient metabolism of plant species has been overlooked for a long time. Only 5% of intracellular water is used for physiological processes in plant species [29,30], such as photosynthesis [31], respiration [32], and nutrient uptake, and transport [33,34], but this can be explained using plant electrophysiology technology. In this experiment, we qualified the intracellular water metabolism of *B. rapa* (Equations (15)–(20)). The IWHC and the WRT were significantly negatively correlated with the *Plasmodiophora* concentration (Table 4), whereas the IWHT was markedly positively correlated. This finding indicates that *B. rapa* responded to *Plasmodiophora* infection by increasing its intracellular water-holding capacity and water translocation rate. It was reported that plants impede water loss and maintain growth by extending the intracellular water-holding time. For example, *Orychophragmus violaceus* (*Ov*) under high-cadmium-level stress suppresses growth and metabolism, reducing the loss of water and other materials by decreasing its cellular water-holding capacity and intracellular water-transfer rate, while increasing its intracellular water-holding time [15]. However, in karst soils, *Ov* adapt to adverse conditions by prolonging the intracellular water-holding time [26], which is consistent with the results of the present study.

Additionally, it has been considered that intracellular nutrient transport is highly dependent on water metabolism in plant species [35,36], so we also qualified the intracellular nutrient metabolism of *B. rapa* at different *Plasmodiophora* infection concentrations (Equations (21)–(25)). The *Plasmodiophora* concentration positively correlated with the UNF, and it was negatively correlated with NTR, NTC, UAF, and NAC of *B. rapa* (Table 5), suggesting that the nutrient translocation rate, translocation capacity, active flow, and active translocation capacity were reduced. This result indicated this plant species replenish nutrient losses by enhancing nutrient translocation under restricted growth [37,38]. Notably, with worsening *Plasmodiophora* infection, *B. rapa* maintained its UNF to reduce the decline in intracellular water content and nutrient metabolism, which shows its adaptive strategy to *Plasmodiophora* infection.

### 4.3. Dissociation of Energy Metabolism and Dielectric Substance Transfer in B. rapa During Plasmodiophora Infection

In previous studies, it has been reported that plant immunity closely relates to cell energy metabolism and the B-type dielectric substance transfer percentage [39], cell energy metabolism positively correlates with plant growth [40,41], while the B-type dielectric substance transfer percentage reflects the distributional characteristics of nutrient transporter proteins of plant cells [42,43]. In this study, we found dissociation between cellular metabolism energy and growth of *B. rapa* during worsening *Plasmodiophora* infection. In Figure 4a, ΔG_R-E_, ΔG_Z-E_, and ΔG exhibited the maximum values at PWB2 and the minimum values at PWB5, but the biomass and photosynthesis continuously declined with worsening *Plasmodiophora* infection. This result is consistent with previous research, showing that in plant species, the photosynthesis and intracellular water and nutrient transfer capacities increased during low-level infection [44,45], but reduced during high-level infection [46,47]. Additionally, we found an interesting result; ∆G was consistent with photosynthesis, but did not consistently decrease (Figure 4b). There was an increasing trend in ∆G of *B. rapa* at PWB2 and PWB4, suggested a transient increase in cellular energy metabolism rather than a sustained decrease during worsening *Plasmodiophora* infection, which revealed this plant species prevented its growth due to continuous inhibitions under environmental stress [48].

In this experiment, we define BPn_R_ as the proportion of extrinsic proteins and BPn_Xc_ as the proportion of binding proteins. We found dissociation between the B-type dielectric substance transfer percentage and growth of *B. rapa* during worsening *Plasmodiophora* infection. As depicted in Figure 5, BPn_R_ and BPn_Xc_ of *B. rapa* exhibited opposite trends, with the BPn_R_ values being higher than the CK, whereas the BPn_Xc_ values were lower than the CK. Furthermore, it demonstrated that BPn_R_ and BPn_Xc_ of *B. rapa* did not exhibit linear increases or decreases. Specifically, the BPn_R_ of PWB4 was the highest, the BPn_Xc_ was the lowest, and the remaining groups did not show significant differences with the CK, resulting in highest extrinsic protein content and the lowest binding protein content. It has found when the pathogen infected, the higher intracellular nutrient loss of the plants enhanced passive transport, leading to an increase in extrinsic proteins, but a decrease in binding proteins on the cell membrane [49,50]. Consequently, we found PWB4 might be the highest immunological concentration of *Plasmodiophora* infection in *B. rapa.*

### 4.4. Immunological Relevance of Electrophysiological Properties of B. rapa During Plasmodiophora Infection

**Figure 7:**
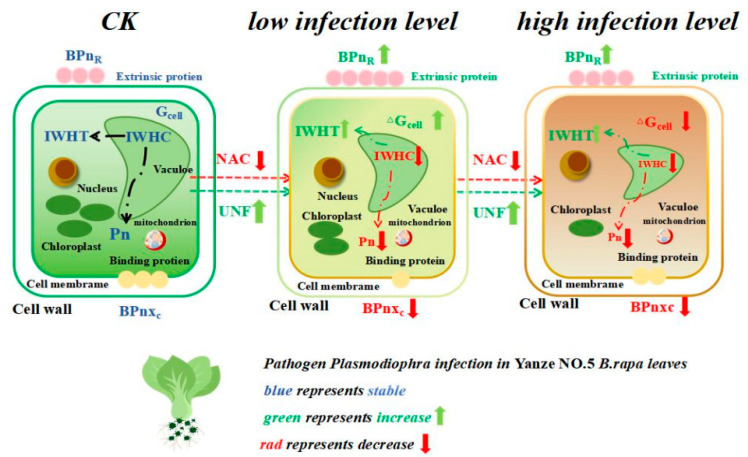
Plant electrophysiological parameters of nutrient immunoregulation in *B. rapa* at different *Plasmodiophora* infection levels. In Figure 7, BPn_R_ denotes B-type dielectric material transfer capacity of _R_, BPn_Xc_ denotes B-type dielectric material transfer capacity of X_C_, IWHC denotes intracellular water-holding capacity, IWHT denotes intracellular water-holding time, G_cell_ denotes unit for cell metabolizable energy, ΔG_cell_ denotes change in unit for cell metabolizable energy, and Pn denotes the photosynthetic rate.

The correlation results indicate that as *Plasmodiophora* infection worsened, the growth of *B. rapa* was inhibited, leading to a decrease in its cellular metabolic energy, as evidenced by a smaller ΔG (Figure 6). Consequently, there were lower values for ΔG, C, the IWHC, the WRT, the NTR, and the NAC. The decreased C of *B. rapa* reduced the vesicle volume, which is critical for maintaining cellular structure and function. In response to these adverse conditions, *B. rapa* increased nutrient transfer through the UAF to sustain its material supply. Several immune mechanisms of plant species infected with *Plasmodiophora* have been shown. On the one hand, plants inhibit nutrient supply by decreasing their energy level. In this study, we found *B. rapa* cells decreased the metabolizable energy level during worsening *Plasmodiophora* infection (Figure 4), supporting this result. On the other hand, plants could strengthen the cell wall, such as increasing lignin and cork deposition to enhance immunity [51,52]. We found that the BPn_R_ of *B. rapa* cells increased during worsening *Plasmodiophora* infection, indicating the promotion of extra-membrane transporter proteins, which contributes to the resistance against *Plasmodiophora* infection [53,54]. Meanwhile, the reduced BPnXc indicated the lower quantity of surface proteins in *B. rapa* cells, leading to decreased intracellular water metabolism during *Plasmodiophor* infection (Figure 7). Hence, *B. rapa* could endure the *Plasmodiophora* infection by maintaining the total nutrient metabolism and minimizing active nutrient transfer and water metabolic activities to conserve energy and resist the infection.

## 5. Conclusions

The results obtained from this study have confirmed that plant electrophysiological techniques can effectively be used to analyze *B. rapa* infected by *Plasmodiophora* (Figure 7). It has been revealed that the plant electrophysiological approach is well aligned with the growth changes in *B. rapa* during infection by *Plasmodiophora*. The degree of *Plasmodiophora* infection increased inversely with growth of *B. rapa*, physiological capacitance, intracellular water metabolism, nutrient transfer capacity, and the total cellular metabolic energy, while it positively correlated with electrical resistance and nutrient transfer capacity. Unlike photosynthesis and overall growth, the unit for cell metabolic energy exhibited a nonlinear change, initially increasing, and then decreasing. Specifically, the BPn_R_ of *B. rapa* increased at low infection levels. However, at high infection levels, the distribution of B-type dielectric material transfer coefficients balanced to sustain its growth. Typically, based on distribution of the B-type dielectric substance transfer percentages, we found PWB4 was the maximal immune concentration, as evidenced by the maximal BPn_R_, with an increasing quantity of extrinsic proteins in the cell membrane, and the minimum BPn_Xc_, with a decreasing quantity of intracellular binding proteins in the *B. rapa* leaves. These findings underscore the potential of plant electrical signals as indicators of *Plasmodiophora* infection, offering a novel, rapid, non-destructive detection method for agricultural disease management.

## Figures and Tables

**Table 1:** Biomass of *B. rapa* infected with different concentrations of *Plasmodiophora*. The data in the graph are presented as the mean ± standard deviation; n is the number of plants per treatment, n = 5. PWB denotes the infection by *Plasmodiophora* of *B. rapa* at different concentrations as follows: CK-0, PWB1-2 × 10^9^, PWB2-4 × 10^9^, PWB3-6 × 10^9^, PWB4-8 × 10^9^, and PWB5-10 × 10^9^ spores/mL. The different lowercase letters a, b, and c in this table denote the significance of differences at *p* < 0.05.

Treatment	Root (g/Plant)	Shoot (g/Plant)	Total (g/Plant)
CK	3.71 ± 0.17 ^a^	8.79 ± 0.16 ^a^	12.49 ± 0.24 ^a^
PWB1	3.16 ± 0.01 ^a^	8.16 ± 0.03 ^a^	11.30 ± 0.26 ^a^
PWB2	2.28 ± 0.05 ^b^	4.71 ± 0.2 ^b^	6.99 ± 0.25 ^b^
PWB3	2.27 ± 0.1 ^b^	3.69 ± 0.1 ^c^	6.96 ± 0.15 ^b^
PWB4	2.05 ± 0.12 ^b^	3.67 ± 0.1 ^c^	5.71 ± 0.19 ^c^
PWB5	2.2 ± 0.11 ^b^	1.39 ± 0.2 ^d^	3.59 ± 0.12 ^d^

**Table 2:** Fitting equations for electrophysiological parameters and clamping force of *B. rapa* infected by *Plasmodiophora*. PWB denotes *B. rapa* infection experiments at different *Plasmodiophora* treatment concentrations of CK-0, PWB1-2 × 10^9^, PWB2-4 × 10^9^, PWB3-6 × 10^9^, PWB4-8 × 10^9^, and PWB5-10 × 10^9^ spores/mL. C indicates capacitance, R indicates resistance, Z indicates impedance, Xc indicates capacitive reactance, and X_L_ indicates inductive reactance. Bonferroni correction was used for all comparisons in Table 2. R^2^ indicates that fitted equations correlate at 0.99, *p* < 0.01 indicates significance of differences at 0.01, and n = 15 shows number of data fitted in each equation.

Treatment	Capacitance/Clamping Force (C/F)	Resistance/Clamping Force (R/F)	Impedance/clamping force (Z/F)
CK	C = 729.48 + 483.97F	R = 0.0875 + 0.0709e^−0.4772F^	Z = 0.0559 + 0.0460e^−0.5090F^
PWB1	C = 483.99 + 280.83F	R = 0.1311 + 0.1214e^−0.5151F^	Z = 0.0850 + 0.0709e^−0.4922F^
PWB2	C = 390.16 + 178.93F	R = 0.1932 +0.1698e^−0.7741F^	Z = 0.1102 + 0.0919e ^−0.6872F^
PWB3	C = 372.02 + 137.29F	R = 0.1889 + 0.1489e^−0.6490F^	Z = 0.1135 + 0.0865e^−0.6183F^
PWB4	C = 315.33 + 220.70F	R = 0.3043 + 0.2631e^−0.6062F^	Z = 0.1558 + 0.1338e^−0.5379F^
PWB5	C = 159.80 + 102.11F	R = 0.6745 + 0.6210e^−0.7561F^	Z = 0.2969 + 0.2589e^−0.6517F^
Treatment	Tolerance/Clamping force (X_C_/F)	Sense resistance/clamping force (X_L_/F)	R^2^/*p*/n
CK	Xc = 0.0728 + 0.0604e^−0.5291F^	X_L_ = 0.13.0 + 0.1121e^−0.4700F^	R^2^ = 0.99, *p* < 0.01, n = 15
PWB1	Xc = 0.1103 + 0.0862e^−0.5340F^	X_L_ = 0.1878 + 0.1555e^−0.5071F^	R^2^ = 0.99, *p* < 0.01, n = 15
PWB2	Xc = 0.1364 + 0.1058e^−0.6591F^	X_L_ = 0.2800 + 0.2335e^−0.7310F^	R^2^ = 0.99, *p* < 0.01, n = 15
PWB3	Xc = 0.1426 + 0.1050e^−0.6146F^	X_L_ = 0.2841 + 0.2177e^−0.6364F^	R^2^ = 0.99, *p* < 0.01, n = 15
PWB4	Xc = 0.1731 + 0.1484e^−0.5027F^	X_L_ = 0.4587 + 0.3966e^−0.6007F^	R^2^ = 0.99, *p* < 0.01, n = 15
PWB5	Xc = 0.3325 + 0.2745e^−0.6522F^	X_L_ = 0.8771 + 0.7810e^−0.7309F^	R^2^ = 0.99, *p* < 0.01, n = 15

**Table 3:** Electrophysiological information inherent in *B. rapa* at different *Plasmodiophora* concentrations. Values in table are expressed as mean ± standard deviation, n = 5; n is number of plants per treatment. PWB denotes *B. rapa* infection experiments at different *Plasmodiophora* treatment concentrations, which were CK-0, PWB1-2 × 10^9^, PWB2-4 × 10^9^, PWB3-6 × 10^9^, PWB4-8 × 10^9^, and PWB5-10 × 10^9^ spores/mL. IC indicates intrinsic capacitance, IR indicates intrinsic resistance, IZ indicates intrinsic impedance, IXc indicates intrinsic capacitive reactance, and IX_L_ indicates inductive reactance. Lowercase letters a, b, c, d, and e in table denote significance difference at 0.05 (*p* < 0.05).

Treatment	IC_P_/pF	IR/MΩ	IZ/MΩ	IX_C_/MΩ	IX_L_/MΩ
CK	729.48 ± 28.48 ^a^	8.75 ± 1.25 ^c^	5.59 ± 0.44 ^e^	7.28 ± 0.29 ^d^	13.70 ± 1.35 ^d^
PWB1	483.99 ± 46.92 ^b^	14.11 ± 1.07 ^c^	8.50 ± 0.18 ^d^	11.03 ± 1.01 ^c^	18.78 ± 4.99 ^d^
PWB2	390.16 ± 28.28 ^c^	19.32 ± 5.18 ^bc^	11.02 ± 0.65 ^b^	13.64 ± 0.95 ^c^	28.00 ± 3.31 ^c^
PWB3	372.02 ± 6.38 ^cd^	18.89 ± 1.64 ^bc^	11.35 ± 0.42 ^c^	14.26 ± 0.24 ^bc^	28.41 ± 1.66 ^c^
PWB4	315.33 ± 61.16 ^d^	30.43 ± 10.83 ^b^	15.58 ± 2.95 ^a^	17.31 ± 3.78 ^c^	45.87 ± 6.01 ^b^
PWB5	159.80 ± 7.48 ^e^	67.45 ± 10.00 ^a^	29.69 ± 0.82 ^a^	33.25 ± 1.57 ^a^	87.71 ± 7.99 ^a^

**Table 4:** Characteristics of intracellular water metabolism in *B. rapa* at different *Plasmodiophora* concentrations. Values in table are expressed as mean ± standard deviation, n = 5; n is number of plants per treatment. PWB denotes *B. rapa* infection experiments at different *Plasmodiophora* concentrations as follows: CK-0, PWB1-2 × 10^9^, PWB2-4 × 10^9^, PWB3-6 × 10^9^, PWB4-8 × 10^9^, and PWB5-10 × 10^9^ spores/mL. IWHC indicates intracellular water-holding capacity, IWUE indicates intracellular water use efficiency, IWHT indicates intracellular water-holding time, and WRT indicates water rate translocation. Lowercase letters a, b, c, d, and e in table indicate significance difference at 0.05 (*p* < 0.05).

Treatment	IWHC	IWUE (10^−2^)	IWHT	WRT
CK	8102.65 ± 211.46 ^a^	6.74 ± 1.11 ^a^	40.67 ± 1.60 ^b^	199.54 ± 12.54 ^a^
PWB1	6160.25 ± 395.00 ^b^	5.01 ± 2.78 ^ab^	41.00 ± 3.08 ^b^	149.96 ± 1.61 ^b^
PWB2	5337.38 ± 256.34 ^c^	3.78 ± 0.23 ^b^	43.13 ± 5.74 ^ab^	124.63 ± 9.92 ^c^
PWB3	5172.47 ± 59.08 ^cd^	2.99 ± 0.80 ^b^	42.22 ± 0.94 ^ab^	122.57 ± 3.86 ^c^
PWB4	4619.06 ± 612.13 ^d^	5.54 ± 1.91 ^ab^	47.92 ± 1.26 ^a^	96.20 ± 10.40 ^d^
PWB5	2944.35 ± 92.05 ^e^	3.91 ± 0.46 ^ab^	47.46 ± 2.95 ^a^	62.13 ± 2.30 ^e^

**Table 5:** Characteristics of nutrient transport in *B. rapa* at different *Plasmodiophora* concentrations. Values in table are expressed as mean ± standard deviation, n = 5, and n is number of plants in each treatment. PWB denotes infection by *Plasmodiophora* of *B. rapa* at different concentrations as follows: CK-0, PWB1-2 × 10^9^, PWB2-4 × 10^9^, PWB3-6 × 10^9^, PWB4-8 × 10^9^, and PWB5-10 × 10^9^ spores/mL. UNF denotes unit for translocation of nutrients. NTR denotes nutrient translocation rate, NTC indicates nutrient translocation capacity, UAF indicates nutrient active flow, and NAC indicates nutrient active translocation capacity. Lowercase letters a, b, c, d, and e in table indicate significant difference between groups (*p* < 0.05).

Treatment	UNF(10^−2^)	NTR	NTC	UAF(10^−2^)	NAC
CK	183.52 ± 15.07 ^b^	199.54 ± 12.54 ^a^	364.95 ± 6.83 ^a^	53.35 ± 3.05 ^ab^	106.71 ± 12.52 ^a^
PWB1	208.21 ± 26,21 ^ab^	149.96 ± 1.61 ^b^	312.11 ± 37.95 ^ab^	62.70 ± 22.82 ^a^	94.21 ± 35.06 ^a^
PWB2	212.14 ± 59.73 ^ab^	124.63 ± 9.92 ^c^	260.45 ± 50.04 ^bc^	49.40 ± 8.63 ^ab^	62.14 ± 15.15 ^b^
PWB3	198.75 ± 11.49 ^ab^	122.57 ± 3.86 ^c^	243.31 ± 6.60 ^bcd^	50.29 ± 2.16 ^ab^	61.70 ± 4.51 ^b^
PWB4	240.68 ± 70.10 ^ab^	96.20 ± 10.40 ^d^	230.62 ± 71.57 ^cd^	37.47 ± 3.10 ^c^	35.83 ± 1.11 ^bc^
PWB5	280.48 ± 42.46 ^a^	62.13 ± 2.30 ^e^	173.70 ± 20.94 ^d^	38.22 ± 5.32 ^b^	23.82 ± 4.21 ^c^

## Data Availability

The original contributions presented in this study are included in the article/Supplementary Material. Further inquiries can be directed to the corresponding authors.

## Supplementary Materials

The following supporting information can be downloaded at: https://www.mdpi.com/article/10.3390/plants14152337/s1, Figure S1: Preparation of conidial suspension; Figure S2: Analysis of plant electrophysiological information; Figure S3: Growth of *Plasmodiophora*-infested *B. rapa* at different concentration.
