# Supplementary material for: Plant Electrophysiological Parameters Represent Leaf Intracellular Water–Nutrient Metabolism and Immunoregulations in Brassica rapa During Plasmodiophora Infection
Source: Plants (Basel). 2025 Jul 29;14(15):2337. doi: 10.3390/plants14152337 (PMC12349594; doi:10.3390/plants14152337)
Supplement: Supplementary file 1 [file plants-14-02337-s001.zip › plants-3742413-supplementary.pdf]

Supplemental materials for

Plant Electrophysiological Parameters Represent Leaf Intracellular Water–Nutrient Metabolism and Immunoregulations in *Brassica rapa* During *Plasmodiophora* Infection

Antong Xia [1,2], Yanyou Wu [2,*], Kun Zhai [1,*], Dongshan Xiang [1], Lin Li [3], Zhanghui Qin [3] and Gratien Twagirayezu [2]

[1] Hubei Key Laboratory of Selenium Resource Research and Biological Application, Hubei Minzu University, Enshi 445000, China; tone1214910327@163.com (A.X.); zk3100@sohu.com (D.X.)

[2] State Key Laboratory of Environmental Geochemistry, Institute of Geochemistry, Chinese Academy of
Sciences, Guiyang 550081, China; tgratien0@gmail.com

[3] Academy of Agricultural Sciences, Enshi Tujia and Miao Autonomous Prefecture, Enshi 445000, China; m19074875200@163.com (L.L.); 18372501196@163.com (Z.Q.)

* Correspondence: wuyanyou@mail.gyig.ac.cn (Y.W.); zk3100@sina.com (K.Z.)

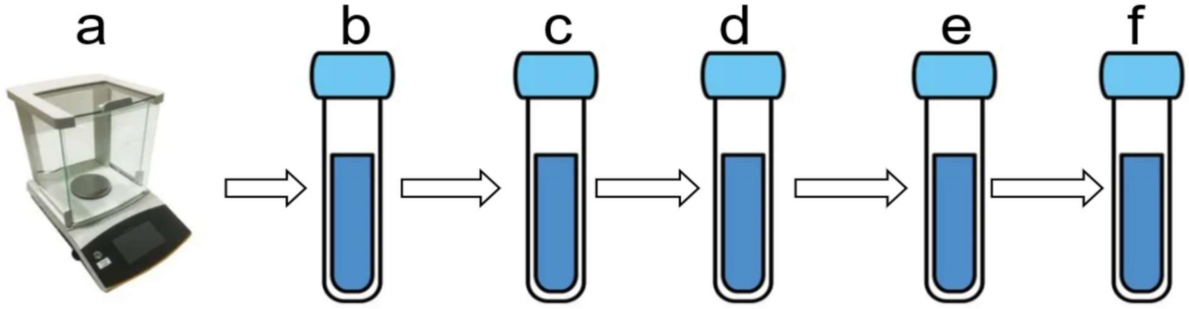

Figure S1 Preparation of Conidial Suspension. 10.00 g of *Plasmodiophora* tissue was weighed and incubated at 24 °C for 5 days (a). The mixture of the supernatant with 30 mL of ultrapure water was centrifuged at 3100 r/min for 15 min, in which the precipitate was mixed with a 50% sucrose solution and centrifuged at 3100 r/min for 10 min (b). Chopped precipitate was added with 50 mL of ultrapure water, churned, filtered, and centrifuged at 500 r/min for 5 min (C). The supernatant was mixed with 30 mL of ultrapure water and centrifuged at 3100 r/min for 10 min; then, the precipitate was combined with a 10% NaCl solution and centrifuged at 3100 r/min for 10 min (d). Finally, the precipitate was mixed with 30 mL of ultrapure water and centrifuged at 3100 r/min for 10 min. We repeated the steps in c and d 2-3 times (e), and we finally prepared a suspension of $10^{\wedge 9}$ spores/mL and stored it at 4 °C (f).

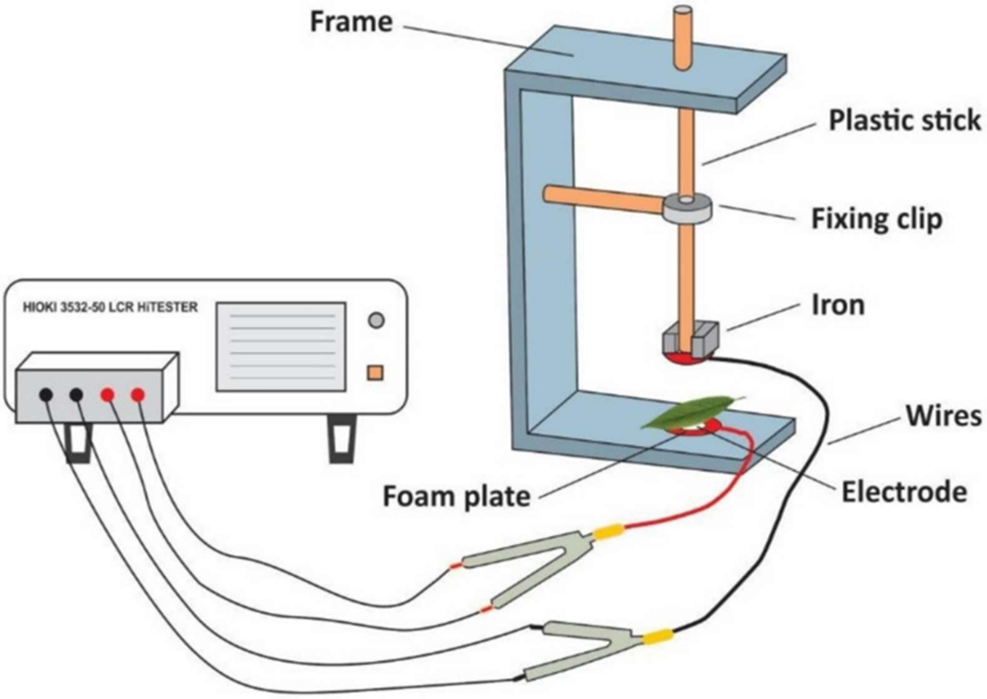

Figure S2 Analysis of plant electrophysiological information.

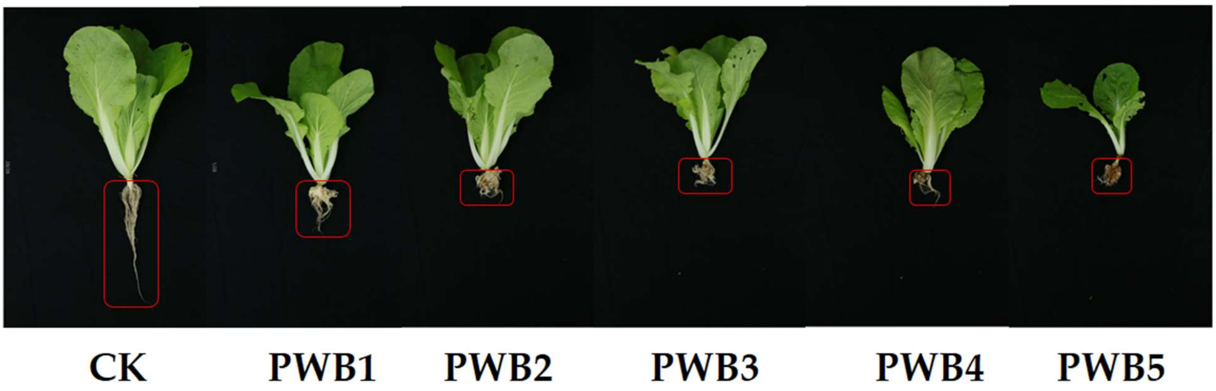

Figure S3 Growth of *Plasmodiophora-infested B. rapa* at different concentrations, which were CK-0, PWB1-2 × 10^9, PWB2-4 × 10^9, PWB3-6 × 10^9, PWB4-8 × 10^9, and PWB5-10 × 10^9 spores/mL.
